# Characterization of the brain virome in human immunodeficiency virus infection and substance use disorder

**DOI:** 10.1371/journal.pone.0299891

**Published:** 2024-04-17

**Authors:** Xin Dang, Barbara A. Hanson, Zachary S. Orban, Millenia Jimenez, Stephen Suchy, Igor J. Koralnik

**Affiliations:** Ken and Ruth Davee Department of Neurology, Northwestern University Feinberg School of Medicine, Chicago, IL, United States of America; Shanghai Public Health Clinical Center, Fudan University, CHINA

## Abstract

Viruses can infect the brain in individuals with and without HIV-infection: however, the brain virome is poorly characterized. Metabolic alterations have been identified which predispose people to substance use disorder (SUD), but whether these could be triggered by viral infection of the brain is unknown. We used a target-enrichment, deep sequencing platform and bioinformatic pipeline named “ViroFind”, for the unbiased characterization of DNA and RNA viruses in brain samples obtained from the National Neuro-AIDS Tissue Consortium. We analyzed fresh frozen post-mortem prefrontal cortex from 72 individuals without known viral infection of the brain, including 16 HIV+/SUD+, 20 HIV+/SUD-, 16 HIV-/SUD+, and 20 HIV-/SUD-. The average age was 52.3 y and 62.5% were males. We identified sequences from 26 viruses belonging to 11 viral taxa. These included viruses with and without known pathogenic potential or tropism to the nervous system, with sequence coverage ranging from 0.03 to 99.73% of the viral genomes. In SUD+ people, HIV-infection was associated with a higher total number of viruses, and HIV+/SUD+ compared to HIV-/SUD+ individuals had an increased frequency of Adenovirus (68.8 vs 0%; p<0.001) and Epstein-Barr virus (EBV) (43.8 vs 6.3%; p=0.037) as well as an increase in Torque Teno virus (TTV) burden. Conversely, in HIV+ people, SUD was associated with an increase in frequency of Hepatitis C virus, (25 in HIV+/SUD+ vs 0% in HIV+/SUD-; p=0.031). Finally, HIV+/SUD- compared to HIV-/SUD- individuals had an increased frequency of EBV (50 vs 0%; p<0.001) and an increase in TTV viral burden, but a decreased Adenovirus viral burden. These data demonstrate an unexpectedly high variety in the human brain virome, identifying targets for future research into the impact of these taxa on the central nervous system. ViroFind could become a valuable tool for monitoring viral dynamics in various compartments, monitoring outbreaks, and informing vaccine development.

## Introduction

Drug use has been linked with the human immunodeficiency virus (HIV) since the beginning of the HIV pandemic. Drug usage is also associated with a higher risk of other viral infections, but little is known about the influence of viruses on substance use disorder (SUD). Therefore, whether viral infections of the brain can trigger an immune, metabolic, regulatory, or chemical alteration that may contribute to drug use in certain individuals, is unknown.

Metagenomic studies in HIV/AIDS and substance use disorder (SUD) research have mainly focused on characterizing HIV clades in various populations and understanding the role of the gut microbiome [1,2].

Conversely, there has been limited emphasis on studying viruses other than HIV and a few selected viral pathogens associated with opportunistic infections. Furthermore, accessing brain areas of interest or cerebrospinal fluid (CSF) samples for targeted viromics studies in the central nervous system (CNS) represents another major obstacle. Therefore, whether the brain virome differs in HIV-infected individuals with and without SUD is unknown.

Deep sequencing (also called *N*e*xt Generation* sequencing; NGS) has played an essential role in identifying viral sequences. However, due to the enormous imbalance between size and abundance of human genomic DNA and RNA and viral nucleic acids, enrichment of viral targets is necessary prior to sequencing. In previous studies, researchers have attempted to concentrate viral particles from input samples through sedimentation [3,4]. However, such methods are restricted to limited cell free specimens, such as feces and plasma, and cannot be used in solid organs. Moreover, viral particle concentration can only identify abundant and actively replicating viruses of a certain density, while species with a low viral load or those that cause latent infections, as well as those that are integrated in the human genome, will be neglected.

To perform a comprehensive evaluation of the human virome, we developed an unbiased, target-enrichment deep-sequencing platform named ViroFind [5]. Because Virofind enriches viral sequences rather than virions, it is appropriate as a general method for human viromic studies using any type of bio-specimen. In a pilot study, ViroFind was capable of enriching viral sequences up to 127-fold from human brain samples compared to deep sequencing alone [5]. This method also allowed us to identify viral sequences in heart samples of patients with myocarditis [6]. We used ViroFind to explore the virome from post-mortem brain samples of individuals with and without HIV and SUD in order to establish a better understanding of which viruses are present in the CNS of people who are affected with these conditions.

## Material and methods

### Clinical samples

A total of 72 frozen prefrontal cortex samples of HIV infected or uninfected individuals with or without SUD were obtained from participating National NeuroAIDS Tissue Consortium (NNTC) centers [7]: Manhattan HIV Brain Bank (MHBB) at Mt Sinai Hospital, NY (n = 39); Brain banks at University of Texas Medical Branch at Galveston (UTMB, n = 20); California NeuroAIDS Tissue Network (CNTN) at University of California, San Diego (UCSD, n= 10); National Neurological AIDS Bank (NNAB) at University of California, Los Angeles (UCLA, n = 3) in different batches, by August 2021. Samples were categorized in four different cohorts by the NNTC staff: HIV+/SUD+, HIV+/SUD-, HIV-/SUD+ and HIV-/SUD-. All samples were processed through the ViroFind pipeline. The NNTC database was accessed on 6/29/2023 to obtain data related to **Tables 1** and **2**. At no time did the authors have access to information that could identify individual participants during or after data collection.

**Table 1:** Study subject demographics.

	Overall (n=72)	HIV+/SUD+ (n=16)	HIV+/SUD- (n=20)	HIV-/SUD+ (n=16)	HIV-/SUD- (n=20)	*p*
Age, years (mean [SD])	52.3 [11.9]	46.2 [8.8]	47.4 [11.7]	59.9 [14.4]	55.9 [7.5]	**<0.001**
Pairwise Tukey’s Post Hoc		46.2 [8.8]	47.4 [11.7]			0.989
		46.2 [8.8]		59.9 [14.4]		**0.002**
		46.2 [8.8]			55.9 [7.5]	**0.048**
			47.4 [11.7]	59.9 [14.4]		**0.005**
			47.4 [11.7]		55.9 [7.5]	0.101
				59.9 [14.4]	55.9 [7.5]	0.684
Sex						
Female, n (%)	27 (37.5)	7 (43.8)	4 (20.0)	8 (50.0)	8 (40.0)	0.261
Male, n (%)	45 (62.5)	9 (56.3)	16 (80.0)	8 (50.0)	12 (60.0)	
Race						
White, n (%)	46 (63.9)	4 (25.0)	16 (80.0)	7 (43.8)	18 (90.0)	**<0.001**
Black/African American, n (%)	21 (29.2)	11 (68.8)	3 (15.0)	6 (37.5)	0 (0.0)	
Other, n (%)	6 (12.0)	1 (6.2)	1 (5.0)	3 (18.7)	2 (10.0)	
Ethnicity						
Hispanic, n (%)	17 (23.6)	2 (12.5)	6 (30.0)	4 (25.0)	5 (25.0)	0.662
Non-Hispanic, n (%)	55 (76.4)	14 (87.5)	14 (70.0)	12 (75.0)	15 (75.0)	

**Table 2:** Frequency of drug used in HIV+/SUD+ and HIV-/SUD+ subjects.

	Urine Screening				Psychiatric Diagnosis			
	Overall (n=31)	HIV+/SUD+ (n=16)	HIV-/SUD+ (n=15)	*p*	Overall (n=19)	HIV+/SUD+ (n=16)	HIV-/SUD+ (n=3)	*p*
No. of substances used by each subject	1[1-3]	3[1.4-4.5]	1[1-1]	**<0.001**	3[1-3]	3[1.5-3.5]	3[3-3]	0.402
(median [IQR])								
Substance % (n)								
Cannabis	31.3 (10)	50.0 (8)	13.3 (2)	0.054	52.6 (10)	62.5 (10)	0 (0)	0.087
Opiates								
Unspecified	53.1 (17)	68.8 (11)	40 (6)	0.156	36.8 (7)	43.8 (7)	0 (0)	0.263
Methadone	28.1 (9)	18.8 (3)	33.3 (5)	0.433	N/A	N/A	N/A	
Oxycodone	3.1 (1)	6.3 (1)	0 (0)	1	N/A	N/A	N/A	
Stimulants								
Unspecified	N/A	N/A	N/A		21.1 (4)	12.5 (2)	66.6 (2)	0.097
Amphetamine/Methamphetamine	9.4 (3)	18.8 (3)	0 (0)	0.226	N/A	N/A	N/A	
Cocaine	40.6 (13)	68.8 (11)	13.3 (2)	**0.003**	63.2 (12)	68.8 (11)	33.3 (1)	0.523
Sedatives								
Unspecified	N/A	N/A	N/A		26.3 (5)	31.3 (5)	0 (0)	0.53
Benzodiazepine	28.1 (9)	43.8 (7)	13.3 (2)	0.113	N/A	N/A	N/A	
Barbiturates	3.1 (1)	6.3 (1)	0 (0)	1	N/A	N/A	N/A	
Hallucinogens								
Unspecified	N/A	N/A	N/A		5.3 (1)	6.3 (1)	0 (0)	1
PCP	6.3 (2)	12.5 (2)	0 (0)	0.484	N/A	N/A	N/A	
EtOH	N/A	N/A	N/A		57.9 (11)	56.3 (9)	66.6 (2)	1
Tricyclic Antidepressants	3.1 (1)	6.3 (1)	0 (0)	1	N/A	N/A	N/A	
Other	9.4 (3)	12.5 (2)	6.7 (1)	1	5.3 (1)	0 (0)	33.3 (1)	0.158

IQR: Interquartile Range; EtOH: Alcohol; PCP: Phencyclidine; N/A: Not available.

### IRB approval

This study was approved by Northwestern University Institutional Review Board STU#: 00211556. All samples used in this study were archival deidentified post-mortem brain samples obtained from the National Neuro-AIDS Tissue Consortium repository (NNTC; https://nntc.org/), and informed consent was not applicable.

### ViroFind design

NNTC prefrontal cortex brain samples of HIV/SUD individuals were kept at -80C prior to processing. Nucleic acid extraction with QIAGEN AllPrep DNA/RNA/miRNA Universal Kit (Cat #: 80224). RNA was reverse transcribed to cDNA with NEBNext Ultra II RNA First Strand Synthesis Module (E7771L) and NEBNext Ultra II Non-Directional RNA Second Strand Synthesis Module (E6111L) from New England Biolabs. Combined genomic DNA (200ng) and cDNA (200ng) from the same sample were then processed through the ViroFind protocol which is an in-solution target-enrichment platform for virus detection and discovery. Pre-hybridization library preparation was performed with the Agilent SureSelect XT HS2 kit (G9983D). Briefly, samples were sonicated to 150-200bp fragments which were ligated to pre-PCR primer adapters on the 3’ ends followed by first round amplification with pre-PCR adapters followed by indexing adapter and post-PCR amplification indexing adapters used at 2X volume to accommodate larger starting nucleic acid concentration (400ng total). All other steps are performed following the kit protocols. NGS library preparation was followed by target enrichment which is achieved through hybridization of viral DNA fragments to biotinylated RNA probes allowing for positive selection of viral sequences. The hybridization was performed as the kit instructed with the following procedure: 95°C 5 min, 65°C 10 min, 65°C 1 min (add 13ul of RNase Blocker solution, probe and SureSelect Fast Hybridization Buffer mixture during this segment), then perform 60 cycles of 65°C 1 min, 37°C 3 sec. After hybridization cycles, the sample was kept at 65°C on hold for a short waiting time or 21°C for overnight hold. The ViroFind probe library targets 561 unique viral genomes with a mean genomic coverage of 81.04% and is comprised of 131,706 unique 125mer probes. Viral species covered include viral taxa known to infect humans and those with zoonotic potential from all Baltimore classifications. Viral species, NCBI accession number, and % of sequence covered by probes are listed in S1 Table. Positively selected viral sequences are isolated with magnetic streptavidin beads and subsequently amplified then isolated through Amplipure magnetic silica. ViroFind libraries were sequenced by NovoGene Paired-end deep sequencing for virus enriched samples (length of reads 150 bp) was performed on the Illumina HiSeq platform.

### Sequencing data analysis

Paired-end deep sequencing for virus enriched samples (length of reads 150 bp) was performed on the Illumina HiSeq platform. Following demultiplexing, each sample had about 20-30 million total reads which were run through the ViroFind analysis pipeline v2.0 [8]. Raw sequencing data was quality checked and filtered for poor quality and low complexity. Reads with overall q-scores <20 and length <50 bp were removed using Skewer (‘-q 20 -I 50 -m pe -z -o’) [9]. Repetitive sequences were removed using PRINSEQ++ (-lc_entropy 60) [10]. Reads which aligned to the reference human genome (hg38, EBV removed) by BWA MEM were subsequently discarded (-M -k 50) [11]. Thereafter, we aligned the remaining reads to a database containing all viral genomes available from the NCBI database (accessed 11/16/2020) using BWA (bwa aln -l 32 -k 2; bwa samse -n 1000). Identified viral regions were matched with gene descriptions corresponding to viral references from NCBI using the BEDOPS program and in-house script [12]. PCR duplicates were marked and removed using the PICARD tool [13]. Finally, the reads from identified viruses were assembled into larger contiguous sequences using SPAdes denovo assembler (-m 10) [14]. Consensus sequence and FASTQ files were generated for reads mapping to different viruses using SAMTOOLS [15].Results for all the samples were evaluated and complex heatmaps were generated using R script as previously described [16–18].

### Curation and thresholding

Data were manually curated as follows: Any viral taxa which included repetitive regions only were removed. Viral taxa which are not known to infect humans, and those with fewer than 10 reads identified were manually assessed through NCBI basic local alignment search tool (blast) to confirm the correct attribution, reads which did not blast to the identified virus were removed.

We conducted a sensitivity analysis to establish an appropriate threshold for our virome data. Initially, viruses identified with fewer than 10 reads were subject to manual confirmation to ensure accuracy. Multiple thresholding analyses were performed, ranging from 1 to 10 reads, to identify the minimum read count at which our qualitative results remained consistent. Notably, our analyses revealed no statistically significant differences between the thresholds set at 5 and 10 reads. Viral taxa with 5 or fewer reads per sample were subsequently excluded.

### Statistical analysis

Summary demographic data, substance use frequency, and presence of each viral taxa are presented as number of patients (percentage/frequency). Normality was determined by Kolmogorov-Smirnov test using GraphPad Prism 9.4.1 (GP). Normally distributed variables are presented as mean (standard deviation/SD); non-normally distributed variables with median (interquartile range/IQR). Between group ages were compared by one-way ANOVA for normally distributed values with Tukey’s post hoc analysis to determine which groups were statistically dissimilar using GP. Fisher’s Exact testing for independence was performed to compare qualitative data for sex, race, ethnicity, SUD usage, and viral presence using either GP or R-Studio for R. Comparisons of non-normally distributed viral burden data was performed using Mann-Whitney U testing on GP. Differences were considered statistically significant at p-values ≤ 0.05.

## Results and discussion

### Samples

The frozen prefrontal cortex samples (n=72) of HIV infected or uninfected individuals with or without SUD were obtained from participating Neuro-AIDS National Tissue Consortium (NNTC) centers [7] in different batches. Samples were categorized in four different cohorts (HIV+/SUD+, HIV+/SUD-, HIV-/SUD+, and HIV-/SUD-), which were processed through the ViroFind deep sequencing library preparation and bioinformatics pipeline obtaining a median of 3.26 (1.2-8.5) million reads per sample following quality and size filtering, the overall median number of quality controlled and curated viral reads per sample was 9 (0.7-465,281) reads per million (rPM).

### Study subject demographics

The demographics of the study subject populations are shown in **Table 1.**

Overall, the average age was 52.3 y, and HIV+ were approximately a decade younger than HIV- individuals.

There was a predominance of males compared to females (62.5 vs 37.5%) without difference between the groups. However, there was a higher frequency of blacks in the SUD+ groups, either HIV+ or HIV-.

The SUD characteristics for SUD+ subjects are shown in **Table 2**.

Attribution of individuals to the SUD+ group was performed by the NNTC staff. Qualitative drug use data was collated from the NNTC files and consisted of multiple urine screening and neuropsychiatric evaluations. Overall, the median number of substances used by SUD+ individuals was 1 and was higher in HIV+ compared to HIV- individuals (3 vs 1; p<0.001). The most frequently used drugs were opiates (53.1%), and only cocaine was more frequently used by HIV+ than HIV- individuals (68.8 vs 13.3%; p = 0.003).

### Viral species detected in the prefrontal cortex of study subjects

**Fig 1:**
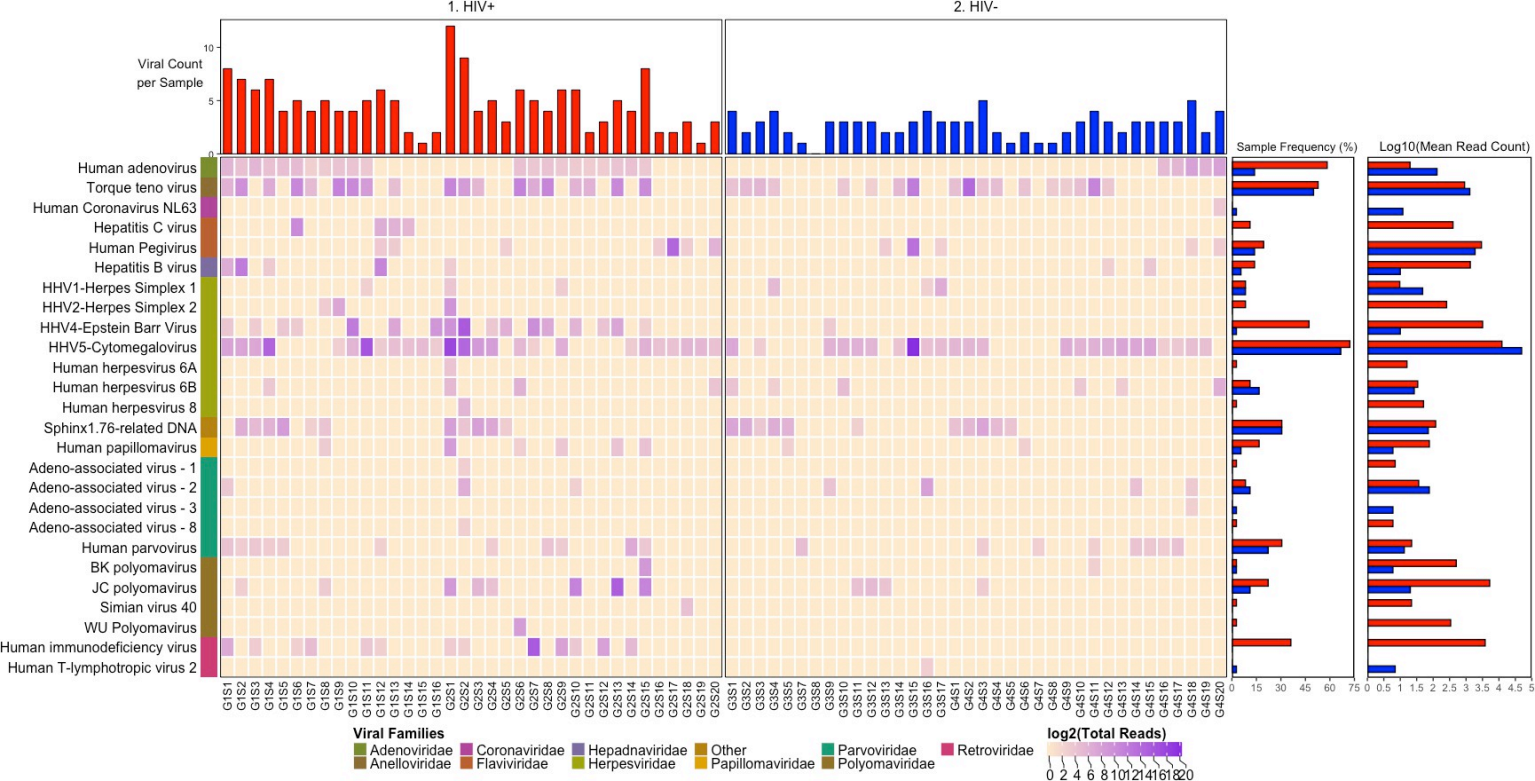
ViroFind bioinformatics pipeline analysis of HIV+ and HIV- individuals. Computed heatmap showing all viral taxa identified by ViroFind in-house pipeline with purple log2 gradient scale indicating the raw number of viral reads. The frequency of each viral species for 36 HIV+ individuals (red) and 36 HIV- individuals (blue) as well as the raw mean read count on a log scale for both groups are shown.

**Fig 2:**
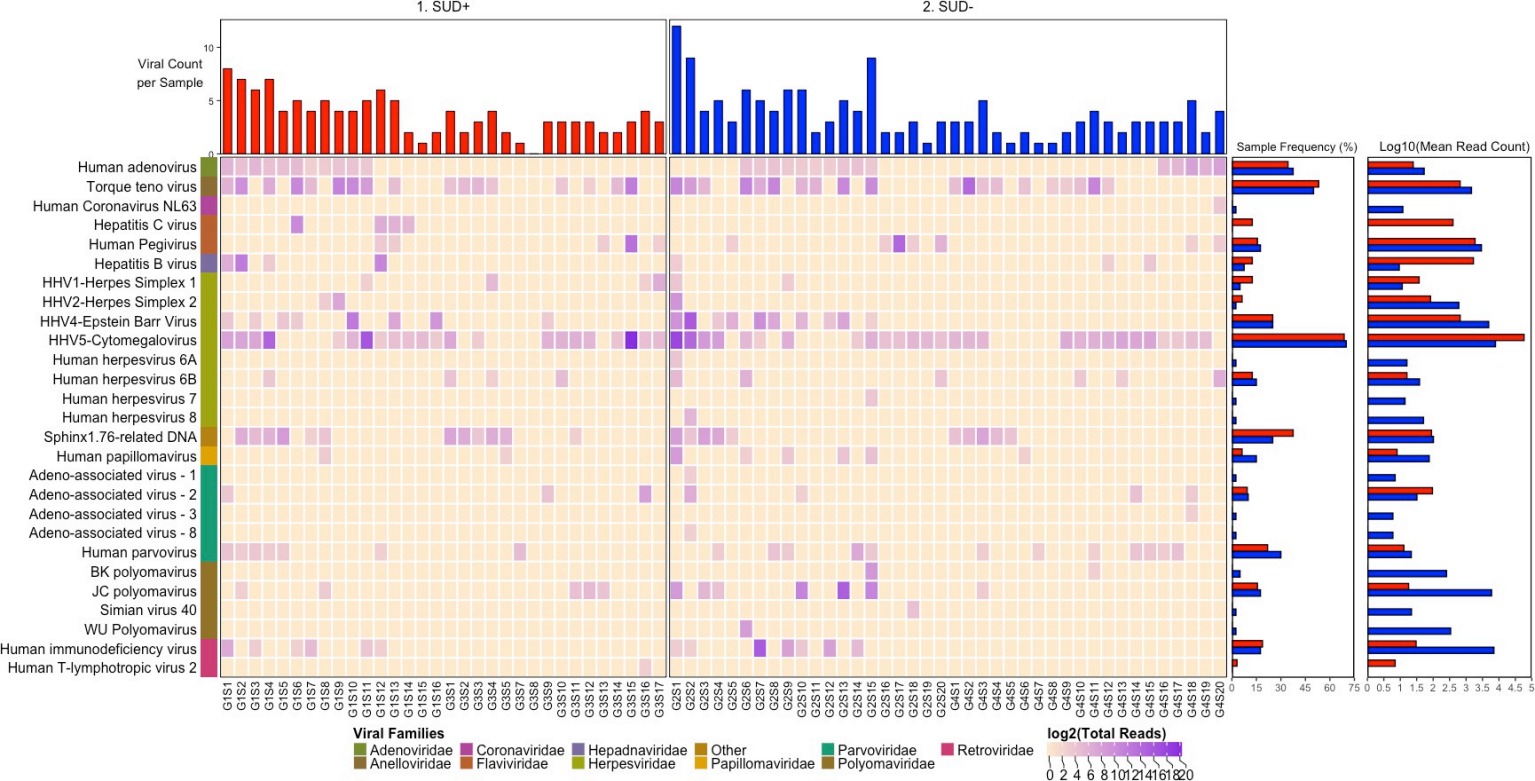
ViroFind bioinformatics pipeline analysis of SUD+ and SUD- individuals. Computed heatmap showing all viral taxa identified by ViroFind in-house pipeline with purple log2 gradient scale indicating the number of raw viral reads. The frequency of each viral species for 32 SUD+ individuals (red) and 40 SUD- individuals (blue) as well as the raw mean read count on a log scale for both groups are shown.

**Fig 3:**
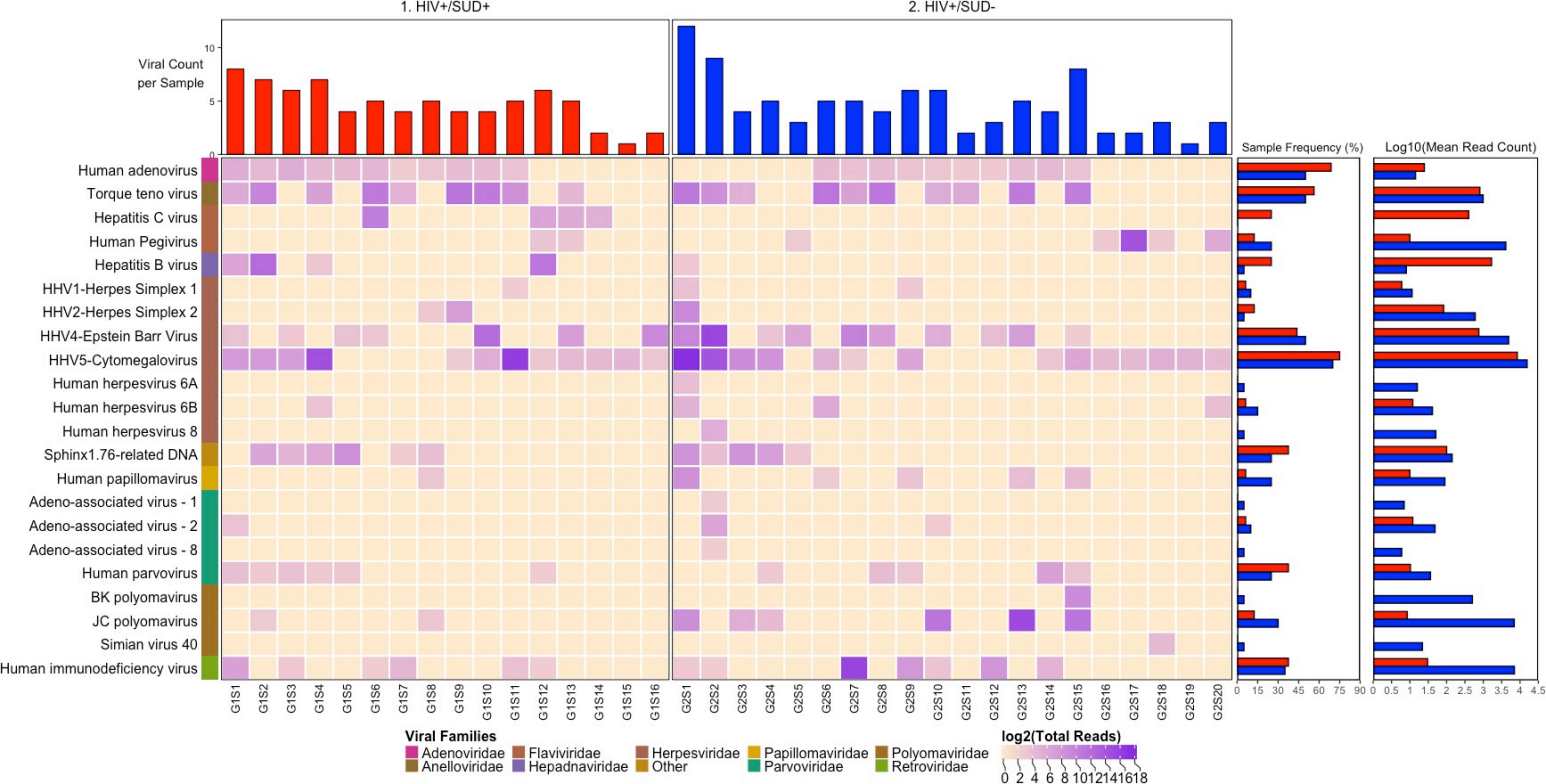
ViroFind bioinformatics pipeline analysis of HIV+/SUD+ and HIV+/SUD- individuals. Computed heatmap showing all viral taxa identified by ViroFind in-house pipeline with purple log2 gradient scale indicating the number of raw viral reads. The frequency of each viral type of 16 HIV+/SUD+ individuals (red) and 20 HIV+/SUD- individuals (blue), as well as the raw mean read count on a log scale for both groups are shown.

**Fig 4:**
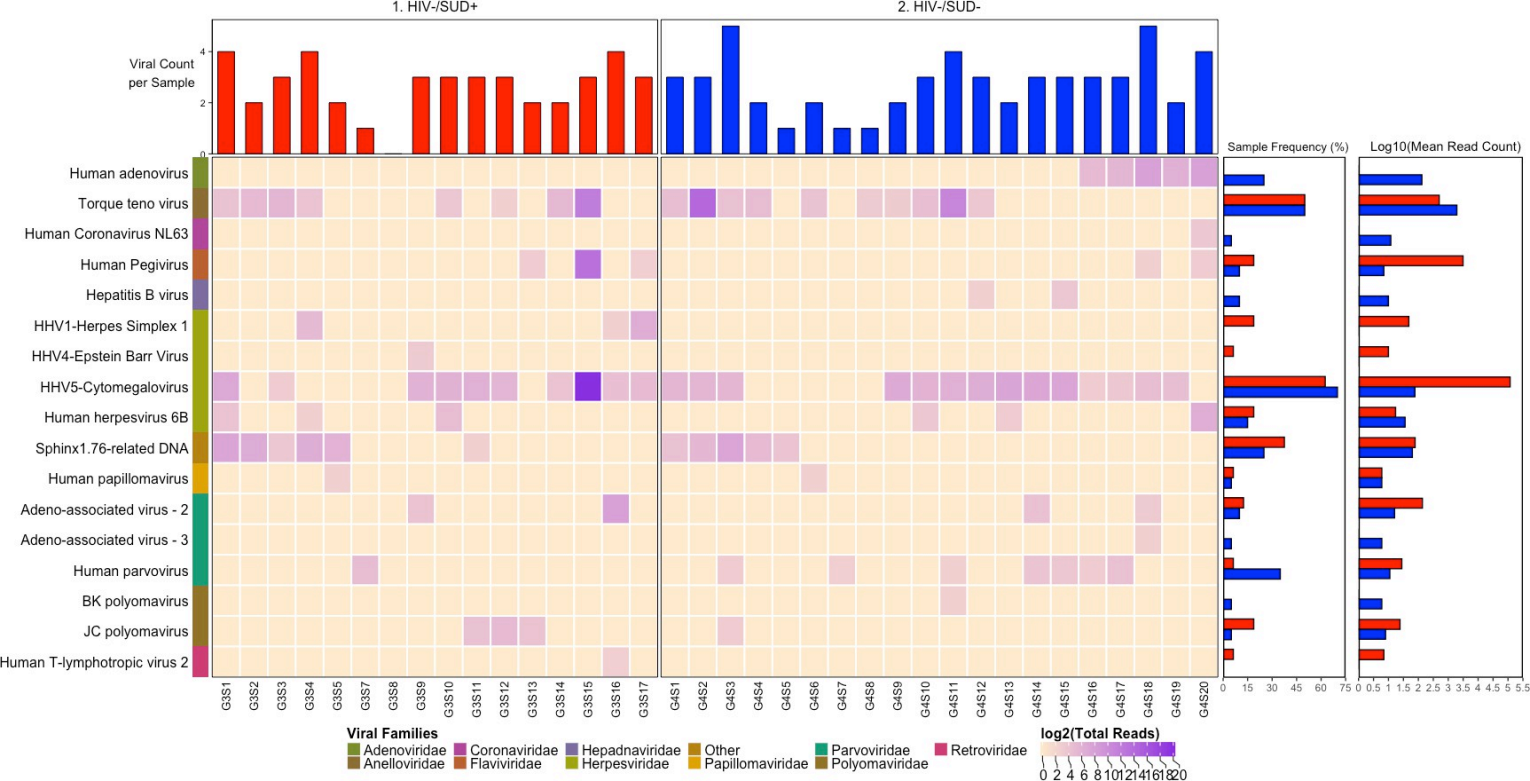
ViroFind bioinformatics pipeline analysis of HIV-/SUD+ and HIV-/SUD- individuals. Computed heatmap showing all viral taxa identified by ViroFind in-house pipeline with purple log2 gradient scale indicating the number of raw viral reads. The frequency of each viral species for 16 HIV-/SUD+ individuals (red) and 20 HIV-/SUD- individuals (blue) as well as the raw mean read on a log scale count for each viral species for both groups are shown.

**Fig 5:**
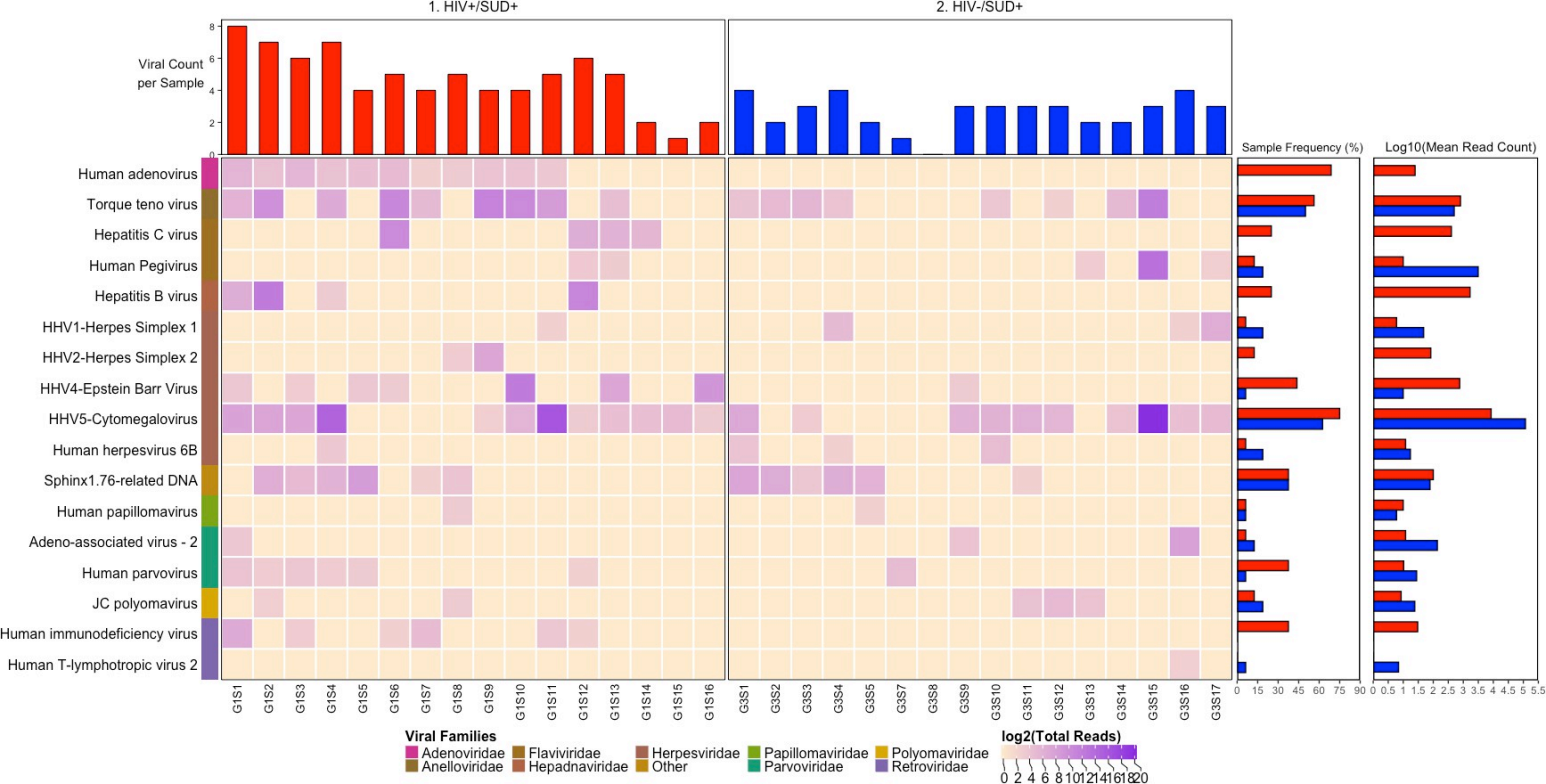
ViroFind bioinformatics pipeline analysis of HIV+/SUD+ and HIV-/SUD+ individuals. Computed heatmap showing all viral taxa identified by ViroFind in-house pipeline with purple log2 gradient scale. Indicating the raw number of viral reads. The frequency of each viral species for 16 HIV+/SUD+ individuals (red) and 16 HIV-/SUD+ individuals (blue) as well as the raw mean read on a log scale count for each viral species for both groups are shown.

**Fig 6:**
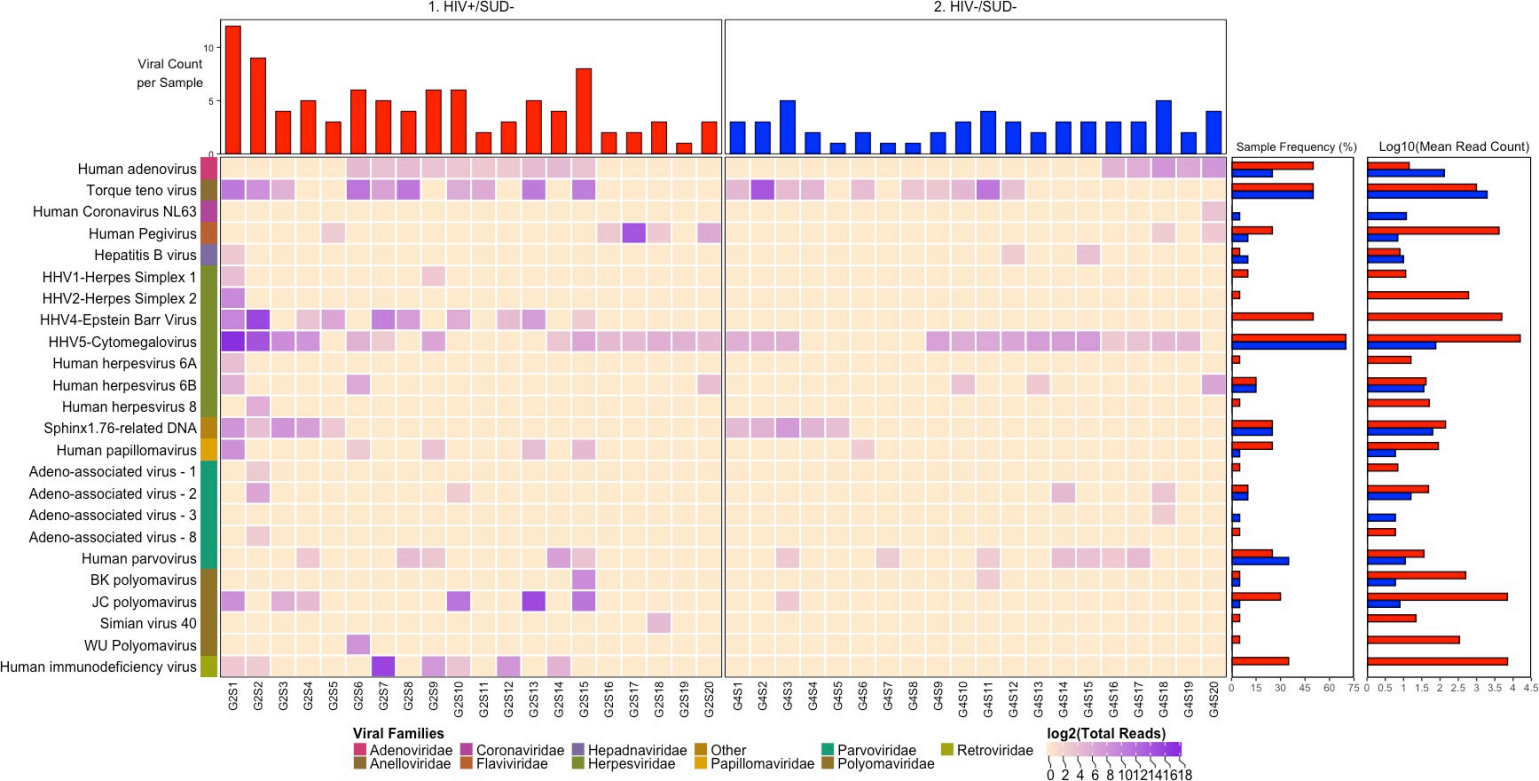
ViroFind bioinformatics pipeline analysis of HIV+/SUD- and HIV-/SUD- individuals. Computed heatmap showing all viral taxa identified by ViroFind in-house pipeline with purple log2 gradient scale indicating the number of raw viral reads. The frequency of each viral species for 20 HIV+/SUD- individuals (red) and 20 HIV-/SUD- individuals (blue) as well as the raw mean read on a log scale count for each viral species for both groups are shown.

We detected nucleic acid from 26 viruses belonging to 11 viral taxa as shown in heatmaps comparing the virome according to HIV infection status (**Fig 1**), SUD status (**Fig 2**), SUD status in HIV+ individuals (**Fig 3**), SUD status in HIV-negative individuals (**Fig 4**), HIV status in SUD+ individuals (**Fig 5**) and HIV status in SUD-negative individuals (**Fig 6**).

We explored the prevalence of viral nucleic acids in HIV + or – individuals (**Fig 1**). The median number of viruses (excluding HIV) detected in the brain sample of each subject was 4 in HIV+ and 3 in HIV- individuals, which was significantly different (p<0.001). In addition, the HIV+ compared to the HIV- group had increased frequency of nucleic acids from Adenovirus (58.3 vs 13.9%; p<0.001), Epstein Barr virus (EBV) (47.2 vs 2.8%; p<0.001) and HIV (36.1 vs 0%; p<0.001). Furthermore, quantitative analyses of the overall viral burden (excluding HIV) per subject showed that it was higher in the HIV+ than in HIV- group (13.5 vs 7.0 viral reads/million [rPM]; p=0.003). Individual viruses with higher burden in HIV+ than in HIV- individuals (excluding HIV) included Adenovirus, and Torque teno virus. (Table 3).

**Table 3:** Viral presence and burden (normalized in rPM) in brain samples from HIV+ and HIV- individuals.

	Viral Presence			Viral Burden		
	36 HIV+	36 HIV-	*p*-Value	36 HIV+	36 HIV-	*p*-Value
	Median viral species per subject (IQR)*			Median viral rPM per subject (IQR)*		
	4 (3-5)	3 (2-3)	<0.001	13.5 (6.1-32.0)	7.0 (4.6-11.1)	0.003
Viral taxa	**n (%)**			**Median rPM (IQR)**		
Human adenovirus	21 (58.3)	5 (13.9)	**<0.001**	5.5 (4-9)	34.6 (24-63)	**<0.001**
Torque teno virus	19 (52.8)	18 (50)	1	94.4 (26-482)	4.4 (3-13)	**<0.001**
Human Coronavirus NL63	0 (0)	1 (2.8)	1		3.1 (N/A)	N/A
Hepatitis C virus	4 (11.1)	0 (0)	0.115	33.8 (27-137)		N/A
Human Pegivirus	7 (19.4)	5 (13.9)	0.753	4 (3-9)	2.6 (2-4)	0.330
Hepatitis B virus	5 (13.9)	2 (5.6)	0.429	23.1 (2-766)	2.2 (2-3)	0.333
Herpes Simplex 1 (HHV1)	3 (8.3)	3 (8.3)	1	2.5 (2-3)	4.6 (4-15)	0.190
Herpes Simplex 2 (HHV2)	3 (8.3)	0 (0)	0.239	49.2 (26-95)		N/A
Epstein Barr Virus (HHV4)	17 (47.2)	1 (2.8)	**<0.001**	32.4 (4-178)	3.1 (N/A)	0.177
Cytomegalovirus (HHV5)	26 (72.2)	24 (66.7)	0.798	17 (8-60)	14.6 (8-20)	0.367
Human herpesvirus 6A	1 (2.8)	0 (0)	1	3.7 (N/A)		N/A
Human herpesvirus 6B	4 (11.1)	6 (16.7)	0.735	6.3 (3-12)	2.7 (2-6)	0.241
Human herpesvirus 7	1 (2.8)	0 (0)	1	11.4 (N/A)		N/A
Human herpesvirus 8	1 (2.8)	0 (0)	1	9 (N/A)		N/A
Sphinx1.76-related DNA	11 (30.6)	11 (30.6)	1	16.1 (5-56)	11.3 (4-18)	0.393
Human papillomavirus	6 (16.7)	2 (5.6)	0.260	6.6 (4-14)	4.6 (4-5)	1
Adeno-associated virus – 1	1 (2.8)	0 (0)	1	1.2 (N/A)		N/A
Adeno-associated virus – 2	3 (8.3)	4 (11.1)	1	3.9 (3-10)	5.2 (5-38)	0.377
Adeno-associated virus – 3	0 (0)	1 (2.8)	1		2.6 (N/A)	N/A
Adeno-associated virus – 8	1 (2.8)	0 (0)	1	1.1 (N/A)		N/A
Human parvovirus	11 (30.6)	8 (22.2)	0.594	3.1 (2-4)	3.8 (2-6)	0.836
BK polyomavirus	1 (2.8)	1 (2.8)	1	409.6 (N/A)	1.4 (N/A)	N/A
JC polyomavirus	8 (22.2)	4 (11.1)	0.343	58.9 (6-1627)	6.3 (4-8)	0.203
Simian virus 40	1 (2.8)	0 (0)	1	10.6 (N/A)		N/A
WU Polyomavirus	1 (2.8)	0 (0)	1	97.1 (N/A)		N/A
Human immunodeficiency virus	13 (36.1)	0 (0)	**<0.001**	7.4 (2-28)		N/A
Human T-lymphotropic virus 2	0 (0)	1 (2.8)	1		3.8 (N/A)	N/A

IQR: Interquartile range; rPM: Reads per million; N/A: Not Applicable

*excluding HIV.

We then explored the prevalence of viral nucleic acids in SUD+ or – individuals (**Fig 2**). The median number of viruses detected in the brain sample of each subject was 3 in both SUD+ and SUD- individuals, HIV inclusive, which was not significantly different. However, the SUD+ compared to the SUD- group had increased frequency of nucleic acids for Hepatitis C virus (HCV) (12.5 vs 0%; p=0.035). Furthermore, quantitative analyses of the overall viral burden per subject showed that there was no significant difference in the SUD+ than in SUD- group (8.6 vs 9.7 rPM; p<0.757). (Table 4).

**Table 4:** Viral presence and burden (normalized in RPM) in brain samples from SUD+ and SUD- individuals.

	Viral Presence			Viral Burden		
	32 SUD+	40 SUD-	*p*-Value	32 SUD+	40 SUD-	*p*-Value
	Median viral species per subject (IQR)			Median viral rPM per subject (IQR)		
	3 (2-4.5)	3 (2-4.5)	0.726	8.6 (5.0-13.9)	9.7 (5.1-17.3)	0.757
Viral taxa	**n (%)**			**Median rPM (IQR)**		
Human adenovirus	11 (34.4)	15 (37.5)	0.810	5.5 (4-8)	9.3 (5-20)	0.194
Torque teno virus	17 (53.1)	20 (50)	0.817	14.9 (4-206)	25.6 (6-456)	0.552
Human Coronavirus NL63	0 (0)	1 (2.5)	1		3.1 (N/A)	N/A
Hepatitis C virus	4 (12.5)	0 (0)	**0.035**	33.8 (27-137)		N/A
Human Pegivirus	5 (15.6)	7 (17.5)	1	4 (4-4)	2.9 (2-9)	0.626
Hepatitis B virus	4 (12.5)	3 (7.5)	0.692	394.4 (18-859)	1.9 (2-2)	0.112
Herpes Simplex 1 (HHV1)	4 (12.5)	2 (5)	0.396	3.9 (3-10)	3 (3-3)	0.817
Herpes Simplex 2 (HHV2)	2 (6.3)	1 (2.5)	0.581	26.2 (15-38)	139.9 (N/A)	0.540
Epstein Barr Virus (HHV4)	8 (25)	10 (25)	1	3.5 (3-114)	34.5 (13-156)	0.143
Cytomegalovirus (HHV5)	22 (68.8)	28 (70)	1	14.3 (9-35)	16.1 (6-36)	0.977
Human herpesvirus 6A	0 (0)	1 (2.5)	1		3.7 (4-4)	N/A
Human herpesvirus 6B	4 (12.5)	6 (15)	1	2.7 (2-4)	6.3 (3-17)	0.241
Human herpesvirus 7	0 (0)	1 (2.5)	1		11.4 (N/A)	N/A
Human herpesvirus 8	0 (0)	1 (2.5)	1		9 (N/A)	N/A
Sphinx1.76-related DNA	12 (37.5)	10 (25)	0.308	13 (5-23)	8.4 (4-41)	0.974
Human papillomavirus	2 (6.25)	6 (15)	0.287	4.1 (4-4)	7 (4-14)	0.617
Adeno-associated virus – 1	0 (0)	1 (2.5)	1		1.2 (N/A)	N/A
Adeno-associated virus – 2	3 (9.4)	4 (10)	1	5.3 (4-72)	4.7 (4-8)	0.860
Adeno-associated virus – 3	0 (0)	1 (2.5)	1		2.6 (N/A)	N/A
Adeno-associated virus – 8	0 (0)	1 (2.5)	1		1.1 (N/A)	N/A
Human parvovirus	7 (21.9)	12 (30)	0.592	2.7 (2-4)	3.8 (3-6)	0.375
BK polyomavirus	0 (0)	2 (5)	0.499		205.5 (103-308)	N/A
JC polyomavirus	5 (15.6)	7 (17.5)	1	5 (4-8)	102.9 (11-1682)	0.104
Simian virus 40	0 (0)	1 (2.5)	1		10.6 (N/A)	N/A
WU Polyomavirus	0 (0)	1 (2.5)	1		97.1 (N/A)	N/A
Human immunodeficiency virus	6 (18.8)	7 (17.5)	1	2.8 (2-6)	17.9 (5-115)	0.353
Human T-lymphotropic virus 2	1 (3.1)	0 (0)	0.444	3.8 (N/A)		N/A

IQR: Interquartile range; rPM: Reads per million; N/A: Not Applicable.

We then explored the prevalence of viral nucleic acids in HIV+/SUD + or HIV+/SUD- individuals (**Fig 3**). There was no significant difference in the median number of viruses detected in the brain sample of each subject between the HIV+/SUD+ group and the HIV+/SUD- group, HIV inclusive. However, the HIV+/SUD+ group compared to the HIV+/SUD- group had an increased incidence of nucleic acids from HCV (25 vs 0%; p=0.010). Furthermore, quantitative analyses of the overall viral burden per subject showed no significant difference between the two groups. (**Table 5**).

**Table 5:** Viral presence and burden (normalized in rPM) in brain samples from HIV+/SUD+ and HIV+/SUD- individuals.

	Viral Presence			Viral Burden		
	16 HIV+/SUD+	20 HIV+/SUD-	*p*-Value	16 HIV+/SUD+	20 HIV+/SUD-	*p*-Value
	Median viral species per subject (IQR)			Median viral rPM per subject (IQR)		
	5 (4-6)	4 (3-6)	0.631	10.8 (4.8-18.3)	15.1 (7.9-25.8)	0.317
Viral taxa	**n (%)**			**Median rPM (IQR)**		
Human adenovirus	11 (68.8)	10 (50)	0.320	5.5 (4-8)	5.3 (4-9)	0.916
Torque teno virus	9 (56.3)	10 (50)	0.749	94.4 (15-356)	255.5 (31-626)	0.391
Hepatitis C virus	4 (25)	0 (0)	**0.031**	33.8 (27-137)		N/A
Human Pegivirus	2 (12.5)	5 (25)	0.426	4.1 (4-4)	3.8 (3-14)	0.846
Hepatitis B virus	4 (25)	1 (5)	0.149	394.4 (18-859)	1.9 (N/A)	0.289
Herpes Simplex 1 (HHV1)	1 (6.25)	2 (10)	1	1.4 (N/A)	3 (3-3)	0.540
Herpes Simplex 2 (HHV2)	2 (12.5)	1 (5)	0.574	26.2 (15-38)	139.9 (N/A)	0.540
Epstein Barr Virus (HHV4)	7 (43.8)	10 (50)	0.749	3.7 (3-157)	34.5 (13-156)	0.262
Cytomegalovirus (HHV5)	12 (75)	14 (70)	1	13.5 (9-48)	21.6 (8-97)	0.777
Human herpesvirus 6A	0 (0)	1 (5)	1		3.7 (N/A)	N/A
Human herpesvirus 6B	1 (6.3)	3 (15)	0.613	3 (N/A)	9 (6-14)	0.371
Human herpesvirus 7	0 (0)	1 (5)	1		11.4 (N/A)	N/A
Human herpesvirus 8	0 (0)	1 (5)	1		9 (N/A)	N/A
Sphinx1.76-related DNA	6 (37.5)	5 (25)	0.483	12.5 (7-21)	46.8 (3-65)	0.784
Human papillomavirus	1 (6.3)	5 (25)	0.196	3.9 (N/A)	9.3 (4-16)	1
Adeno-associated virus – 1	0 (0)	1 (5)	1		1.2 (N/A)	N/A
Adeno-associated virus – 2	1 (6.25)	2 (10)	1	2.8 (N/A)	10 (7-13)	0.540
Adeno-associated virus – 8	0 (0)	1 (5)	1		1.1 (N/A)	N/A
Human parvovirus	6 (37.5)	5 (25)	0.483	2.5 (2-3)	4 (3-10)	0.055
BK polyomavirus	0 (0)	1 (5)	1		409.6 (N/A)	N/A
JC polyomavirus	2 (12.5)	6 (30)	0.257	2.8 (2-3)	836.8 (37-1738)	0.067
Simian virus 40	0 (0)	1 (5)	1		10.6 (N/A)	N/A
WU polyomavirus	0 (0)	1 (5)	1		97.1 (N/A)	N/A
Human immunodeficiency virus 1	6 (37.5)	7 (35)	1	2.8 (2-6)	17.9 (5-115)	0.353

IQR: Interquartile range; rPM: Reads per million; N/A: Not Applicable.

We then qualitatively explored the prevalence of viral nucleic acids in HIV-/SUD+ and HIV-/SUD- individuals (**Fig 4**). There was no significant difference in the median number of viruses or of any individual virus detected in the brain sample of each subject between the HIV-/SUD+ and HIV-/SUD- groups. Furthermore, quantitative analyses of the overall viral burden or of the burden of individual viruses showed no significant differences between the two groups (**Table 6**).

**Table 6:** Viral presence and burden (normalized in rPM) in brain samples from HIV-/SUD+ and HIV/SUD- individuals.

	Viral Presence			Viral Burden		
	16 HIV-/SUD+	20 HIV-/SUD-	*p*-Value	16 HIV-/SUD+	20 HIV-/SUD-	*p*-Value
	Median viral species per subject (IQR)			Median viral rPM per subject (IQR)		
	3 (2-3)	3 (2-3)	0.976	8.3 (5.1-11.9)	6.0 (4.1-10.5)	0.624
Viral taxa	**n (%)**			**Median rPM (IQR)**		
Human adenovirus	0 (0)	5 (25)	0.053		34.6 (24-63)	N/A
Torque teno virus	8 (50)	10 (50)	1	3.8 (3-10)	5.3 (3-13)	0.625
Human Coronavirus NL63	0 (0)	1 (5)	1		3.1 (N/A)	N/A
Human Pegivirus	3 (18.8)	2 (10)	0.637	3.6 (3-1855)	2.3 (2-2)	0.773
Hepatitis B virus	0 (0)	2 (10)	0.492		2.2 (2-3)	N/A
Herpes Simplex 1 (HHV1)	3 (18.8)	0 (0)	0.078	4.6 (4-15)		N/A
Epstein Barr Virus (HHV4)	1 (6.3)	0 (0)	0.444	3.1 (N/A)		N/A
Cytomegalovirus (HHV5)	10 (62.5)	14 (70)	0.729	15.8 (9-19)	12.9 (6-22)	0.838
Human herpesvirus 6B	3 (18.8)	3 (15)	1	2.4 (2-5)	2.9 (2-13)	0.663
Sphinx1.76-related DNA	6 (37.5)	5 (25)	0.483	13 (5-21)	5.6 (5-11)	0.648
Human papillomavirus	1 (6.3)	1 (5)	1	4.4 (N/A)	4.8 (N/A)	N/A
Adeno-associated virus – 2	2 (12.5)	2 (10)	1	71.6 (38-105)	4.7 (5-5)	0.245
Adeno-associated virus – 3	0 (0)	1 (5)	1		2.6 (N/A)	N/A
Human parvovirus	1 (6.3)	7 (35)	0.053	12.7 (N/A)	3.8 (2-4)	0.190
BK polyomavirus	0 (0)	1 (5)	1		1.4 (N/A)	N/A
JC polyomavirus	3 (18.8)	1 (5)	0.303	7.6 (6-8)	0.9 (N/A)	0.371
Human T-lymphotropic virus 2	1 (6.3)	0 (0)	0.444	3.8 (N/A)		N/A

IQR: Interquartile range; rPM: Reads per million; N/A: Not Applicable.

We then explored the prevalence of viral nucleic acids in HIV+/SUD+ or HIV-/SUD+ individuals (**Fig 5**). There was a significantly higher median number of viruses (excluding HIV) in the HIV+/SUD+ group compared to the HIV-/SUD+ group (4 vs 3; p=0.005). In addition, the HIV+/SUD+ group had increased frequency of nucleic acids from Adenovirus (68.8 vs 0%, p<0.001) and EBV compared to the HIV-/SUD+ group (43.8 vs 6.3%; p=0.037). Furthermore, quantitative analyses of the overall viral burden per subject (excluding HIV) showed that there were no significant differences overall between the HIV+/SUD+ than in HIV-/SUD+ groups. Of all viruses detected, Torque teno virus had a significantly higher viral burden in HIV+/SUD+ than in HIV-/SUD+ individuals (**Table 7**).

**Table 7:** Viral presence and burden (normalized in rPM) in brain samples from HIV+/SUD+ and HIV-/SUD+ individuals.

	Viral Presence			Viral Burden		
	16 HIV+/SUD+	16 HIV-/SUD+	*p*-Value	16 HIV+/SUD+	16 HIV-/SUD+	*p*-Value
	Median viral species per subject (IQR)*			Median viral rPM per subject (IQR)*		
	4 (3.75-5)	3 (2-3)	0.005	13.5 (5.3-38.0)	8.3 (5.1-11.9)	0.126
Viral taxa	**n (%)**			**Median rPM (IQR)**		
Human adenovirus	11 (68.8)	0 (0)	**<0.001**	5.5 (4-8)		N/A
Torque teno virus	9 (56.3)	8 (50)	1	94.4 (15-356)	3.8 (3-10)	**0.024**
Hepatitis C virus	4 (25)	0 (0)	0.101	33.8 (27-137)		N/A
Human Pegivirus	2 (12.5)	3 (18.8)	1	4.1 (4-4)	3.6 (3-1855)	0.773
Hepatitis B virus	4 (25)	0 (0)	0.101	394.4 (18-859)		N/A
Herpes Simplex 1 (HHV1)	1 (6.3)	3 (18.8)	0.600	1.4 (N/A)	4.6 (4-15)	0.371
Herpes Simplex 2 (HHV2)	2 (12.5)	0 (0)	0.484	26.2 (15-38)		N/A
Epstein Barr Virus (HHV4)	7 (43.8)	1 (6.3)	**0.037**	3.7 (3-157)	3.1 (N/A)	0.383
Cytomegalovirus (HHV5)	12 (75)	10 (62.5)	0.704	13.5 (9-48)	15.8 (9-19)	0.921
Human herpesvirus 6B	1 (6.3)	3 (18.8)	0.600	3 (N/A)	2.4 (2-5)	1
Sphinx1.76-related DNA	6 (37.5)	6 (37.5)	1	12.5 (7-21)	13 (5-21)	0.936
Human papillomavirus	1 (6.3)	1 (6.3)	1	3.9 (N/A)	4.4 (N/A)	N/A
Adeno-associated virus – 2	1 (6.3)	2 (12.5)	1	2.8 (N/A)	71.6 (38-105)	0.540
Human parvovirus	6 (37.5)	1 (6.3)	0.083	2.5 (2-3)	12.7 (N/A)	0.211
JC polyomavirus	2 (12.5)	3 (18.8)	1	2.8 (2-3)	7.6 (6-8)	0.149
Human immunodeficiency virus 1	6 (37.5)	0 (0)	**0.018**	2.8 (2-6)		N/A
Human T-lymphotropic virus 2	0 (0)	1 (6.3)	1		3.8 (N/A)	N/A

IQR: Interquartile range; rPM: Reads per million; N/A: Not Applicable

*excluding HIV.

Finally, we explored the prevalence of viral nucleic acids in HIV+/SUD- or HIV-/SUD- individuals (**Fig 6**). There was a significant difference in the median number of viruses (excluding HIV) detected in the brain sample of each subject in HIV+/SUD- compared to the HIV-/SUD- groups (4 vs 3, p=0.035). In addition, the HIV+/SUD- compared to the HIV-/SUD- group had increased frequency of EBV (50 vs 0%; p<0.001). Furthermore, quantitative analyses showed an increase in the overall viral burden (excluding HIV) per subject in the HIV+/SUD- compared to the HIV-/SUD- group (14.0 vs 6.0 median viral rPM; p=0.008). Adenovirus viral burden higher in the HIV-/SUD- group relative to HIV+/SUD- individuals (p=0.003), whereas HIV+/SUD- had a higher burden of Torque teno viruses (p=0.017). (**Table 8**).

**Table 8:** Viral presence and burden (normalized in rPM) in brain samples from HIV+/SUD-and HIV-/SUD- individuals.

	Viral Presence			Viral Burden		
	20 HIV+/SUD-	20 HIV-/SUD-	*p*-Value	20 HIV+/SUD-	20 HIV-/SUD-	*p*-Value
	Median viral species per subject (IQR)*			Median viral rPM per subject (IQR)*		
	4 (2.75-5)	3 (2-3)	0.035	14.0 (7.3-28.3)	6.0 (4.1-10.5)	0.008
Viral taxa	**n (%)**			**Median rPM (IQR)**		
Human adenovirus	10 (50)	5 (25)	0.191	5.3 (4-9)	34.6 (24-63)	**0.003**
Torque teno virus	10 (50)	10 (50)	1	255.5 (31-626)	5.3 (3-13)	**0.017**
Human Coronavirus NL63	0 (0)	1 (5)	1		3.1 (N/A)	N/A
Human Pegivirus	5 (25)	2 (10)	0.407	3.8 (3-14)	2.3 (2-2)	0.333
Hepatitis B virus	1 (5)	2 (10)	1	1.9 (N/A)	2.2 (2-3)	1
Herpes Simplex 1 (HHV1)	2 (10)	0 (0)	0.487	3 (3-3)		N/A
Herpes Simplex 2 (HHV2)	1 (5)	0 (0)	1	139.9 (N/A)		N/A
Epstein Barr Virus (HHV4)	10 (50)	0 (0)	**<0.001**	34.5 (13-156)		**N/A**
Cytomegalovirus (HHV5)	14 (70)	14 (70)	1	21.6 (8-97)	12.9 (6-22)	0.346
Human herpesvirus 6A	1 (5)	0 (0)	1	3.7 (N/A)		N/A
Human herpesvirus 6B	3 (15)	3 (15)	1	9 (6-14)	2.9 (2-13)	0.663
Human herpesvirus 7	1 (5)	0 (0)	1	11.4 (N/A)		N/A
Human herpesvirus 8	1 (5)	0 (0)	1	9 (N/A)		N/A
Sphinx1.76-related DNA	5 (25)	5 (25)	1	46.8 (3-65)	5.6 (5-11)	0.676
Human papillomavirus	5 (25)	1 (5)	0.182	9.3 (4-16)	4.8 (N/A)	1
Adeno-associated virus – 1	1 (5)	0 (0)	1	1.2 (N/A)		N/A
Adeno-associated virus – 2	2 (10)	2 (10)	1	10 (7-13)	4.7 (5-5)	1
Adeno-associated virus – 3	0 (0)	1 (5)	1		2.6 (N/A)	N/A
Adeno-associated virus – 8	1 (5)	0 (0)	1	1.1 (N/A)		N/A
Human parvovirus	5 (25)	7 (35)	0.731	4 (3-10)	3.8 (2-4)	0.33
BK polyomavirus	1 (5)	1 (5)	1	409.6 (N/A)	1.4 (N/A)	N/A
JC polyomavirus	6 (30)	1 (5)	0.091	836.8 (37-1738)	0.9 (N/A)	0.211
Simian virus 40	1 (5)	0 (0)	1	10.6 (N/A)		N/A
WU Polyomavirus	1 (5)	0 (0)	1	97.1 (N/A)		N/A
Human immunodeficiency virus	7 (35)	0 (0)	**0.008**	17.9 (5-115)		N/A

IQR: Interquartile range; rPM: Reads per million; N/A: Not Applicable

*excluding HIV.

Overall, the median % (IQR) sequence coverage of the viral genomes for all samples was 3.22% (0.84-8.07), with a range of 0.03-99.73%. Sequence coverage of > 90% are shown in S1 Fig and have been deposited in GenBank. To determine if the presence of HCV and HBV in the brain was associated with known infection by those viruses, we reviewed the available serologic and virologic data in the NNTC database. Of 7 individuals who had detectable HBV in their brain by ViroFind, only two had been tested for HBV infection during their lifetime, including 1 with positive and 1 with negative results. Conversely, of 65 individuals with undetectable HBV by ViroFind, 35 had been tested for HBV infection during their lifetime, including 3 with positive and 32 with negative results. Therefore, there was no association between ViroFind testing of prefrontal cortex samples and previously documented HBV infection (Fisher’s exact test p= 0.207).

However, of 4 individuals who had detectable HCV by ViroFind, all had been tested for HCV infection during their lifetime and found to be positive. Conversely, of 68 individuals with undetectable HCV by ViroFind, 39 had been tested for HCV infection during their lifetime including 17 who were positive and 22 who were negative. Therefore, there was an association between ViroFind prefrontal cortex results and previously documented HCV infection (Fisher’s exact test p=0.049).

### Qualitative and quantitative diversity of the brain virome

Although the brain has long been considered a sterile environment, our unbiased method for virus detection reveals an unexpectedly large number of DNA and RNA viral sequences in the prefrontal cortex of individuals with and without HIV-infection or SUD. Although none of the samples came from individuals with known active viral encephalitis, we detected several viral species with known pathogenic potential in the nervous system (eg. Herpes Simplex, Cytomegalovirus, JC polyomavirus), species with known pathogenic potential outside of the nervous system (eg Adenovirus, HCV, HBV, BK polyomavirus), as well as species with no defined pathogenic potential (eg Torque teno virus, Pegivirus, adeno-associated viruses). We even detected sequences of Simian Polyomavirus 40 in a few samples, a virus thought to be restricted to monkeys, which was inoculated unwittingly to millions of people in the US in the late fifties as a contaminant of polio vaccines [19].

In addition, ViroFind provides a semi-quantitative evaluation of the viral burden, allowing comparisons between groups for the entire virome as well as individual viruses. The qualitative and quantitative diversity of viral species discovered in our study validates ViroFind as a suitable method for detection of DNA and RNA viruses of all sizes in human samples. Indeed, ViroFind can detect large viral species such as EBV with a genome of ~172 Kbp, that is potentially integrated in the human genome, as well as small and non-integrated polyomaviruses with genomes of ~10 Kbp. Conversely, studies limited to the screening of human genomic databases for viruses may be biased towards DNA viruses only, which may be lost if the sequencing library preparation is tailored for exome research. Furthermore, RNA or DNA viruses may be lost with poly-A selection that is biased for mRNA, which is commonly used in RNAseq transcriptome analysis [20].

### Association of HIV-infection and the brain virome

Overall, brain samples from HIV+ individuals harbored a greater number of viral taxa than those of HIV- people, and HIV-infection was frequently associated with an increased total viral burden. It is possible that the immunosuppression associated with HIV was responsible for this increase in viral load in the brain. In addition, viruses found more frequently in HIV+ individuals included Adenovirus and EBV, which have been associated with brain diseases [21,22]. Torque teno virus (TTV) had a higher viral burden in HIV+ people. Of interest, TTV has an increased viremia in HIV+ patients [23].

### Association of SUD and the brain virome

While brain samples from SUD+ people did not harbor a greater number of viral taxa compared to SUD- individuals, HCV was found more frequently present in brain tissue from SUD+ people. Although this virus has been associated with SUD [24], its presence in the brain is tantalizing. Indeed, HCV-associated neurologic disorder most frequently involves the peripheral nervous system, causing peripheral neuropathy because of proliferation of B lymphocytes and production of cryoglobulins [25]. However, central nervous system manifestations independent from hepatic encephalopathy secondary to liver cirrhosis have been described. Potential mechanisms include infection of astrocytes and microglia [26,27]. Of note, another hepatitis virus that was not expected to be found in the brain is HBV. Although HBsAg and HBV DNA have been detected in the CSF of HBV-infected patients with neuro-psychiatric manifestations, whether the virus itself or immune reaction to the virus is responsible for these symptoms remains unclear [28]. Limited available serologic and virologic data during the study subjects’ lifetime showed an association with ViroFind results for HCV but not HBV in the brain. Future studies will be necessary to understand the full range of the compartmentalization of those viruses in the body.

### Combined association of HIV and SUD and the brain virome

Subgroup analyses allowed us to further explore the combined association of HIV and SUD in the brain virome. The association of HIV in SUD+ individuals was an increase in the total number of viruses and frequency of adenovirus and EBV as well as an increase in the TTV viral burden compared to HIV-/SUD+ group. In addition, the SUD phenotype in HIV+ individuals was associated with an increased frequency of HCV compared to the HIV+/SUD- group. Altogether, the variety of the brain virome, and increased frequency of some viral species and viral burden associated with HIV and SUD is enticing. However, confirmation of these findings and whether viral species detected by ViroFind are latent or replicating, and the characterization of their precise location and cellular localization in the brain parenchyma will require further investigations.

### Potential role of virus infection of the brain in SUD

While we have identified brain virome associations with both HIV and SUD status in this study, it is conceivable that viruses may also be associated with the etiology of SUD. This study provides a first exploration into which viruses are present in the brain parenchyma of a matched cohort of people with and without HIV and SUD. Our data shows that many viruses can reach the CNS and be detected in the prefrontal cortex. While the cellular localization of those viruses requires further study, it is possible that they remain latent, thereby escaping detection from the immune system. It is therefore possible that recurrent reactivations in the setting of immunosuppression or inflammation may trigger an immune, metabolic, regulatory, or chemical imbalance that may contribute to drug use in certain individuals. This has been observed to occur through alterations of dopamine metabolism, as demonstrated with Japanese Encephalitis Virus [29], Borna Disease Virus [30], and HIV-Tat expression [31,32]. Activation of Extracellular Regulated Kinases (ERK) has been implicated in SUD [33] and HIV reactivation [34] and may be induced by other viral infections. Indeed, many viruses have been shown to affect the ERK pathway [35,36]. Certain viruses have also been shown to activate Phospho-Lipase C (PLC), an enzyme that increases drug seeking behavior when it is upregulated in the ventral tegmental area of the midbrain [29,37]. Alternatively, viral integration into the human genome may act as modulator of gene expression in diverse ways such as dopamine regulation, even in absence of active replication. This is the case for integration of HERV-K LTR in RASGRF2 gene, which is more frequent in IV drug users than controls [38]. The frequency and location of viral integration in the human genome will require further study. The data generated herein provide viral targets for future study to determine whether any of the identified taxa produce similar effects in CNS resident cells which might contribute to SUD.

### Limitations

This study, as with all currently used viral detection methods, has limitations. Unlike bacterial species, which have conserved genetic regions for targeted 16S rRNA amplification, viral genomes are not only too diverse for a similar amplification strategy but also harbor genomes encoded on DNA and RNA. Therefore, designing whole virome identification strategies depends on NGS from both DNA and RNA samples mixed with host nucleic acids which remain far more abundant than viral nucleic acids due to the lack of conserved amplification targets on viral sequences. NGS strategies are rapidly expanding to increased sequencing density which allows a greater read depth per sample; however these new chemistries have shown increased misattribution of reads from sequencing errors and through index hopping [39–43]. Limited misattribution of reads between samples does not qualitatively affect genomic and transcriptomic analyses but can dramatically affect the observations made in metagenomics studies like whole virome sequencing. Thresholding by read number or by regions covered as well as dual-index sequencing can be used to improve the confidence in the qualitative results from previous work. We have elected to threshold on the basis of read numbers, though other strategies should continue to be developed.

We have analyzed the resulting data both qualitatively and semi-quantitatively, however we acknowledge that genome length and secondary structure, PCR efficiency, GC content, and host homology may all confound the number of reads obtained for each individual viral taxon. Consequently, establishing a viral titer from this data remains challenging. Nonetheless, our findings indicate that an increase in the prevalence of viral nucleic acids and the number of viral reads correlates with a higher starting viral load in the sample. In other words, samples with more reads likely had a greater initial viral burden compared to those with fewer reads from the same viral species. Moreover, sequencing based viromics, such as NGS, ViroFind, or viral amplification, is limited in it’s ability to define the status of infection. The presence of viral nucleic acids may be an indication of an active or latent infection but may also be caused by partial sequences/inactivated viral components contained in exosomes or maintained episomally rather than as an indication of localized infection, These and other sequencing based viromics surveys can inform viral targets for further tropism and culturing experiments and are therefore an important first step.

We examined one single brain region, the prefrontal cortex, and it is possible that the composition of the virome differs in other cortical areas and in the white matter, or in the cerebellum, brain stem and spinal cord. Of specific relevance in SUD, brain samples from the ventral tegmentum including the nucleus accumbens, were not available for study. Serologic and virologic data for HCV and HBV was not available from all study subjects, and therefore, correlations with ViroFind data for those two viruses are limited. While ViroFind may allow for detection of viral variants or potentially, novel species bearing some homology to known viruses, the genome coverage of each virus may vary depending on the viral load, from a few reads up to close to the entire genome. Nevertheless, even detection of minute amounts of any virus by ViroFind can inform targeted detection using species-specific PCR primers of various part of its genome, followed by further analysis using Sanger sequencing.

## Conclusions

ViroFind allows for unbiased identification of DNA and RNA viruses in all human clinical samples. In addition to characterizing the entire human virome in various conditions and diseases, ViroFind could also be used as a surveillance tool for emergence of viral variants and, potentially, novel viruses in various populations. As the world slowly emerges from the COVID-19 pandemic, ViroFind could become a valuable tool for monitoring viral dynamics in various compartments, monitoring outbreaks, and informing vaccine development.

## Supporting information

S1 Fig Coverage plot of sequences with greater than 90% coverage. Integrated Genome Viewer (IGV) coverage traces for viral species identified with coverage of greater than 90% of the genome length. Study ID number including group designation (G1: HIV+/SUD+; G2: HIV+/SUD-; G3: HIV-/SUD+; and G4: HIV-/SUD-), coverage breadth in percentage of reference genome covered, and depth range for nucleotide coverage are shown. A) JCV sequences aligned to reference NC_001699.1 from three Group 2 (HIV+/SUD-) subjects. B) HBV reads aligned to reference NC_003977.2 from two Group 1 (HIV+/SUD+) subjects. C) HIV reads aligned to reference NC_00182.1 from one Group 2 subject. D) BKV reads aligned to reference NC_001538.1 from one Group 2 subject. E) Sphinx 1.76 episomal DNA reads aligned to reference number LK931492.1 from one Group 1 and one Group 4 (HIV-/SUD-) subject. F) AAV-2 reads aligned to reference NC_001401.2 from a Group 3 (HIV-/SUD+) subject. Colored lines in coverage trace show nucleotide changes from the indicated reference sequence (A: Green; C: Blue; T: Red; and G: Orange).
(TIF)

S1 Table ViroFind probe coverage. Table showing the NCBI accession number for each reference sequence, the viral name, total genome length, genome length which is covered by ViroFind probes, and the calculated percent of the genome which is covered by the probes.
(XLSX)
