# Supplementary figures and images for: Characterization of the brain virome in human immunodeficiency virus infection and substance use disorder
Source: PLoS One. 2024 Apr 17;19(4):e0299891. doi: 10.1371/journal.pone.0299891 (PMC11023569; doi:10.1371/journal.pone.0299891)

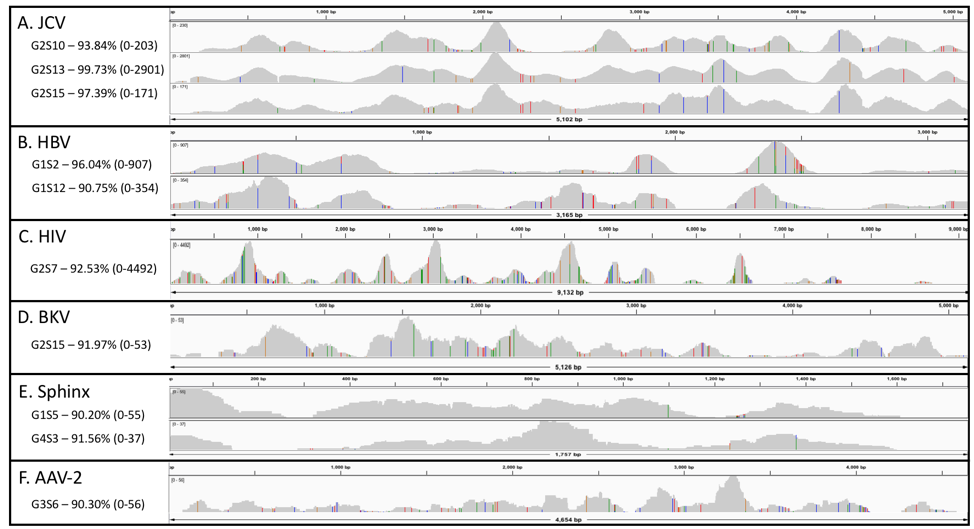

Supplement: S1 Fig — Integrated Genome Viewer (IGV) coverage traces for viral species identified with coverage of greater than 90% of the genome length. Study ID number including group designation (G1: HIV+/SUD+; G2: HIV+/SUD-; G3: HIV-/SUD+; and G4: HIV-/SUD-), coverage breadth in percentage of reference genome covered, and depth range for nucleotide coverage are shown. A) JCV sequences aligned to reference NC_001699.1 from three Group 2 (HIV+/SUD-) subjects. B) HBV reads aligned to reference NC_003977.2 from two Group 1 (HIV+/SUD+) subjects. C) HIV reads aligned to reference NC_00182.1 from one Group 2 subject. D) BKV reads aligned to reference NC_001538.1 from one Group 2 subject. E) Sphinx 1.76 episomal DNA reads aligned to reference number LK931492.1 from one Group 1 and one Group 4 (HIV-/SUD-) subject. F) AAV-2 reads aligned to reference NC_001401.2 from a Group 3 (HIV-/SUD+) subject. Colored lines in coverage trace show nucleotide changes from the indicated reference sequence (A: Green; C: Blue; T: Red; and G: Orange). (TIF) [file pone.0299891.s001.tif]
